# Effects of Donor Cell Types on the Development of Bovine Embryos Using Cytoplasm Injection Cloning Technology

**DOI:** 10.3390/ijms22115841

**Published:** 2021-05-29

**Authors:** Lianguang Xu, Seok-Hwan Song, Muhammad Idrees, Ayman Mesalam, Myeong-Don Joo, Tabinda Sidrat, Yiran Wei, Kyeong-Lim Lee, Wenfa Lu, Il-Keun Kong

**Affiliations:** 1 Division of Applied Life Science (BK21 Four), Department of Animal Science, Gyeongsang National University, Jinju 52828, Korea; xulianguang428@gmail.com (L.X.); idrees1600@gmail.com (M.I.); jmd1441@gmail.com (M.-D.J.); tabindasidrat06@gmail.com (T.S.); weiyiran1230@gmail.com (Y.W.); 2 The King Kong Corp. Ltd., Gyeongsang National University, Jinju 52828, Korea; siwd2002@gmail.com (S.-H.S.); 0920-0728@hanmail.net (K.-L.L.); 3 Institute of Agriculture and Life Science, Gyeongsang National University, Jinju 52828, Korea; 4 Department of Theriogenology, Faculty of Veterinary Medicine, Zagazig University, Zagazig 44519, Egypt; aymanmesalam@gmail.com; 5 Division of Animal Reproduction and Breeding, Department of Animal Science, Jilin Agricultural University, Changchun 130118, China; wenfa2004@163.com

**Keywords:** cytoplasm injection cloning technology, adult fibroblasts, embryonic cells, bovine embryo

## Abstract

Cytoplasm injection cloning technology (CICT) is an efficient technique for evaluating the developmental potential of cloned embryos. In this study, we investigated the effects of donor cell type on the developmental potential and quality of cloned bovine embryos. Adult fibroblasts (AFs) and embryonic cells (ECs) were used as donor cells to clone bovine embryos using CICT. We initially used AF cells to develop cloned embryos and then cultured the cloned day-8 blastocysts for 10 days to obtain ECs as donor cells for second embryo cloning. We found that the bovine blastocysts cloned using AF cells had significantly reduced developmental rates, embryo quality, and ratios of inner cell mass (ICM) to the total number of cells compared to those using ECs as donor cells. Furthermore, there were significant differences in the DNA methyltransferase-, histone deacetylation-, apoptosis-, and development-related genes at the blastocyst stage in embryos cloned from AFs compared to those in embryos cloned from ECs. Our results suggest that using ECs as donor cells for nuclear transfer enhances the quantity and quality of cloned embryos. However, further investigation is required in terms of determining pregnancy rates and developing cloned embryos from different donor cell types.

## 1. Introduction

Cloning refers to the processes used to produce the exact genetic replica of a biological entity. Somatic cell nuclear transfer (SCNT) is the most common method for obtaining cloned animals, where the somatic cells are transferred into recipient enucleated oocytes [1]. SCNT is an important tool for treating infertility and preserving endangered species but is a controversial technique as it is uncontrollable due to various risk factors, such as high rates of embryonic loss, placental abnormalities, postnatal mortalities, or survival for more than a short period of time (1–5%) [2,3,4]. Abnormal genome reprogramming is one of the basic factors of SCNT failure and using extra cytoplasm can play a significant role in the normal reprogramming of the donor cell [5,6].

Cytoplasm injection cloning technology (CICT) is a type of SCNT, where extra cytoplasm is injected into the enucleated oocyte with the donor cell, which improves the quality of cloned embryos [7]. Several studies have identified that CICT is more efficient than the traditional SCNT approach and has been successfully used to clone several livestock animals, such as cattle, cats, and mice [7,8,9]. Several studies have focused on this new technique, which can be of great practical value. Although CICT has been successfully used to some extent and offers a new route for SCNT, it does not completely eliminate the problems faced by SCNT-cloned embryos, such as incomplete nucleus reprogramming, chromosome remodeling failure, and embryonic genomic activation delay. One of the most critical elements in CICT is the donor cell type, which significantly affects nuclear transfer efficiency and reconstructed embryo development [10,11,12]. Several donor somatic cells, such as mammary gland cells [13], granular cells [14], cumulus cells [15,16], oviduct epithelial cells [16,17], skin fibroblasts [18], and others [19], have been used in SCNT. Considerable differences have been found in the ability of different cell lines and cell types to be reprogrammed and in the developmental competence of cloned embryos produced from them. Further, it is still unclear which cell type is the most efficient for SCNT.

In this study, we used adult fibroblasts (AFs) and embryonic cells (ECs) as donor cells to analyze the quality of cloned bovine embryos developed using CICT. We found that the quality and quantity of EC-derived embryos (EC-CICT) was significantly higher than those of AF-derived embryos (AF-CICT). Furthermore, the epigenetics and embryo genomics in the EC-CICT-cloned blastocysts group were not significantly different from those in the normal IVF group. Thus, in the present study, we compared the effectiveness of donor cell types on the developmental potential and blastocyst quality of cloned bovine embryos using the CICT.

## 2. Results

### 2.1. Isolation, Culture and Characterization of Donor Cells

**Figure 1:**
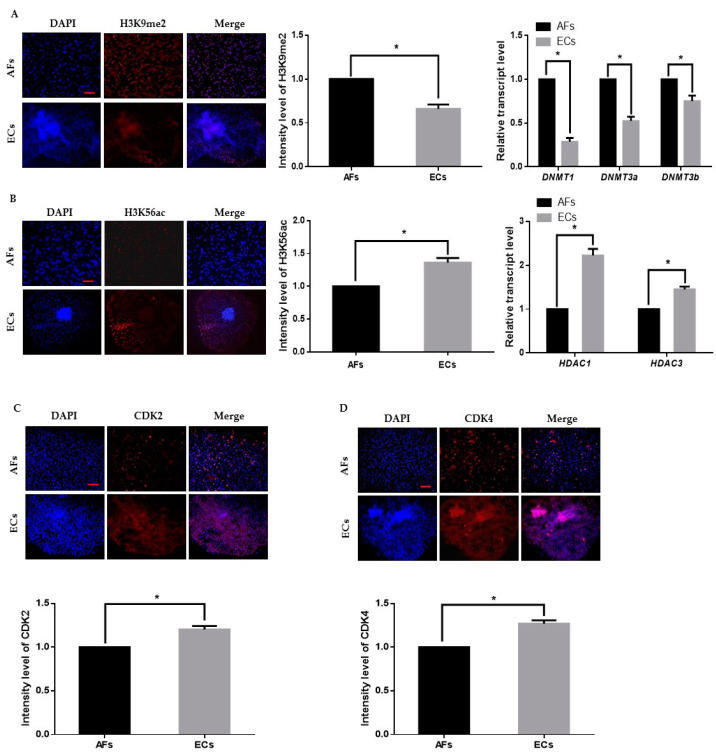
Epigenetic staining of donor cells. (**A**) Representative immunofluorescence images of H3K9me2 in donor cells. Donor cells were stained for H3K9me2 (red) and DNA (DAPI, blue). Bar = 100 µm. Quantification of fluorescence intensity in donor cells. Quantitative real-time polymerase chain reaction (qRT-PCR) results for DNMTs (*DNMT1*, *DNMT3a*, *DNMT3b*) in donor cells. (**B**) Representative immunofluorescence images of H3K56ac in donor cells. Donor cells were stained for H3K56ac (red) and DNA (DAPI, blue). Bar = 100 µm. Quantification of the fluorescence intensity in donor cells. Quantitative real-time polymerase chain reaction (RT-qPCR) results for HDACs (*HDAC1*, *HDAC3*) in donor cells. (**C**,**D**) Representative immunofluorescence images of CDK2 and CDK4 in donor cells. Donor cells were stained for CDK2 and CDK4 (red) and DNA (DAPI, blue). Bar = 100 µm. Quantification of fluorescence intensity in donor cells. Labeling intensity was expressed relative to that of the AFs (set as 100%). The data are from three independent experiments and are means ± SEM (* *p* < 0.05).

We first cultured and transferred AFs to the enucleated oocytes to obtain cloned day-8 blastocysts. Thereafter, we removed the zona pellucida with acid Tyrode’s solution and placed 2–3 blastocysts per culture insert in 8-well IbiTreat µ-plates (Ibidi GmbH; Martinsried, Germany) with ECs culture media at 37 °C with 5% CO_2_. Epigenetic dynamics play an important role in embryonic genome activation and preimplantation embryonic development. We identified that H3K9me2 expression in AFs was significantly higher than that in the ECs, which indicates transcriptional repression in the AFs (Figure 1A). We also analyzed H3K56ac expression in AF and ECs and observed that H3K56ac is highly expressed in the ECs (Figure 1B). As H3K56ac is involved in the cell cycle, we also analyzed the expression levels of cyclin dependent kinase 2 (CDK2) and 4 (CKD4) (Figure 1C,D). The results showed that the expression level of both cell cycle-related proteins was significantly higher in ECs than in AFs. These results suggest that ECs are favorable for reprogramming enucleated oocytes.

### 2.2. Donor Cell Transfer and Development of Cloned Preimplantation Bovine Embryos

**Figure 2:**
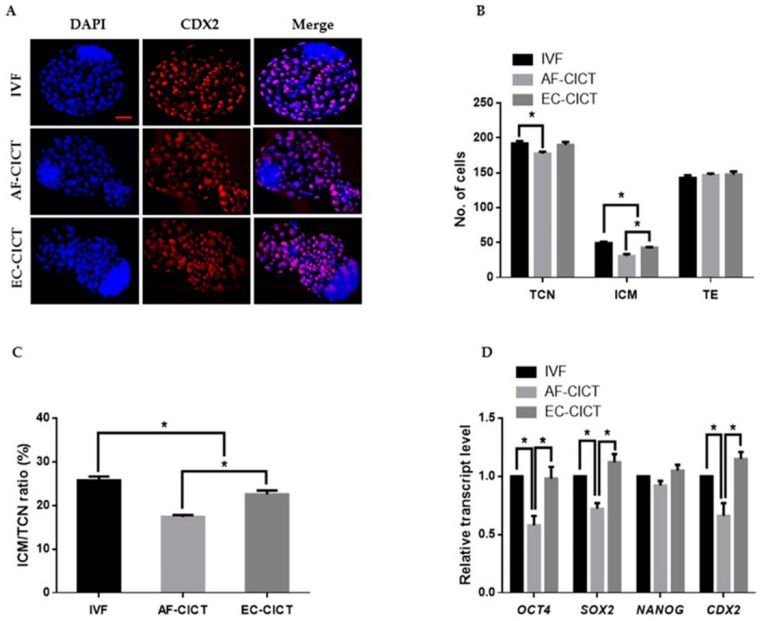
Effect of donor cell types on the developmental competence of bovine CICT embryos. (**A**) Representative immunofluorescence images of CDX2/DAPI in blastocysts. Embryos were stained for CDX2 (red) and DNA (DAPI, blue). Bar = 100 µm. Quantification of the (**B**) total cell number (TCN), inner cell mass (ICM) and trophectoderm (TE) cell numbers, and (**C**) ICM/TCN ratios (*n* = 10 per group). (**D**) Quantitative real-time polymerase chain reaction (RT-qPCR) results for pluripotency-related genes in blastocysts (*n* = 5 per group).

After characterizing the donor cells, the ECs were collected from the culture plate and transferred to enucleated oocytes using CICT. The EC-CICT blastocysts had significantly higher CDX2 positive cells than the AF-CICT blastocysts (Figure 2A); however, there was no significant difference in the number of CDX2 positive cells between EC-CICT and normal IVF blastocysts. Furthermore, the fusion and cleavage rates were similar between AF-CICT and EC-CICT (*p* > 0.05, Table 1). The percentage of EC-CICT 8–16 cell embryos and blastocysts (68.6 ± 1.7 and 32.8 ± 0.8%, respectively) was significantly greater (*p* < 0.05) than that of AF-CICT 8–16 cell embryos and blastocysts (61.9 ± 3.1 and 29.4 ± 1.2%, respectively) and significantly lower (*p* < 0.05) than that of IVF 8–16 cell embryos and blastocysts (77.3 ± 0.9 and 36.6 ± 0.4%, respectively). Further, the total number of cells (TCN), ICM, and trophectoderm (TE) cells was significantly different (Figure 2B; Table S2). By comparing the ICM and TCN ratios, we found that AF-CICT blastocysts had significantly lower ICM than IVF and EC-CICT blastocysts (Figure 2C). To further confirm this, we analyzed the ICM-related genes, such as *OCT4*, *NANOG*, and *SOX2*, and found that their expression was not significantly different between the IVF and EC-CICT blastocysts (Figure 2D). The above results suggest that donor cells significantly affect the embryos cloned using CICT.

### 2.3. Analaysis of Apoptotic Pathways and Dead Cells

**Figure 3:**
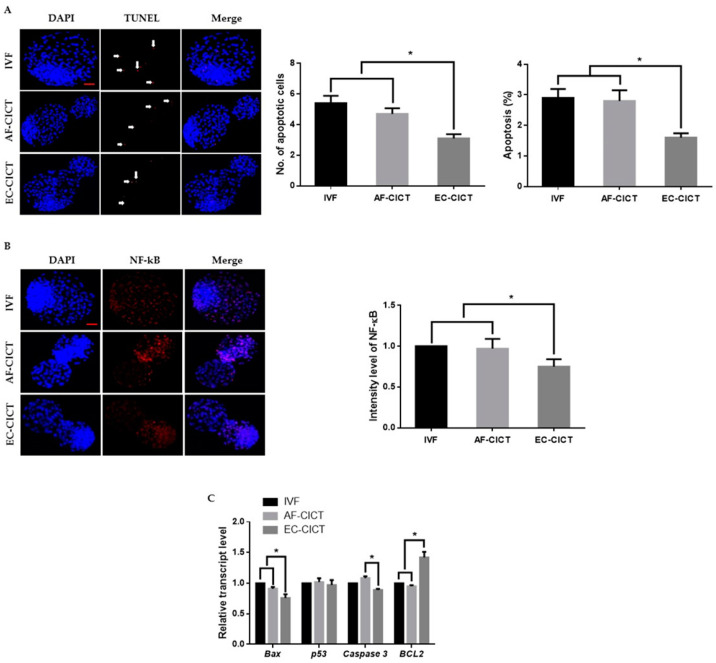
Incidence of apoptosis in blastocysts. (**A**) Terminal deoxynucleotidyl transferase dUTP nick end-labeling (TUNEL) assay of blastocysts. Embryos were stained for TUNEL (red, white arrow) and DNA (DAPI, blue). Bar = 100 µm. Quantification of the number and proportion of apoptotic cells (*n* = 10 per group). (**B**) Representative immunofluorescence images of NF-κB in CICT embryos at the blastocyst stage. Embryos were stained for NF-κB (red) and DNA (DAPI, blue). Bar = 100 µm. Quantification of fluorescence intensity at the blastocyst stage (*n* = 10 per group). (**C**) RT-qPCR results for apoptosis-related genes in blastocysts (*n* = 5 per group). Labeling intensity was expressed relative to that of the IVF group (set as 100%). The data are from three independent experiments and are means ± SEM (* *p* < 0.05).

One of the most critical shortcomings of cloning is the number of dead cells and activated apoptotic pathways in cloned embryos. We, therefore, analyzed the apoptotic cells in the AF-CICT, EC-CICT, and IVF using the terminal deoxynucleotidyl transferase dUTP nick-end labelling (TUNEL) assay. As shown in Figure 3A, Table S3, the apoptotic index was significantly higher (*p* < 0.05) in the AF-CICT than in the EC-CICT (4.6 ± 0.4 vs. 3.3 ± 0.4) but was not significantly different (*p* > 0.05) from that in the IVF (4.6 ± 0.4 vs. 5.4 ± 0.5). By further analyzing the phosphorylated NF-κB expression (Figure 3B), we found that p-NF-kB was highly nuclear localized in the AF-CICT compared to the EC-CICT and IVF. To further confirm the decreased apoptosis level in the EC-CICT, we examined the expression of several apoptosis-related genes, such as *caspase3*, and *Bax*, and observed that their expression level was significantly higher in the AF-CICT than the EC-CICT (Figure 3C). These results suggest that the nuclear transfer of AFs reduces the quality of bovine blastocysts.

### 2.4. Epigenetic Dynamics in Day-8 Bovine Blastocysts

**Figure 4:**
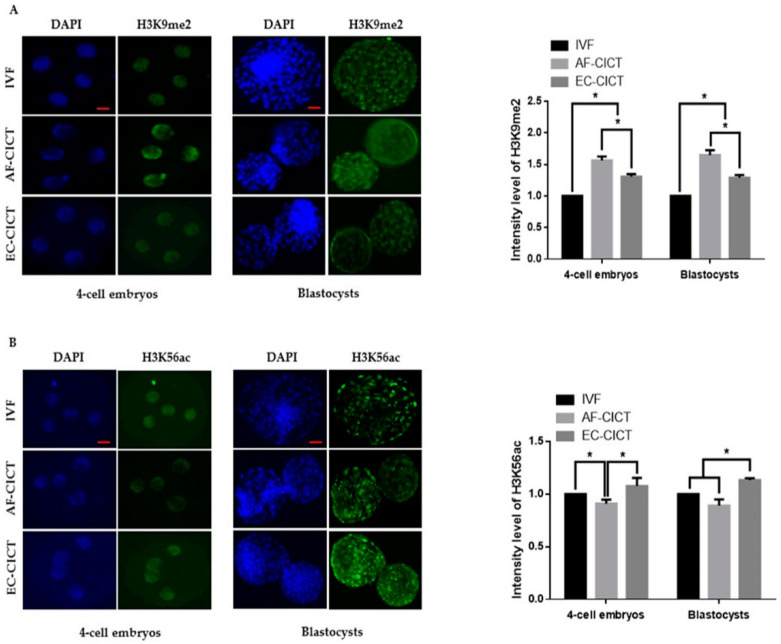
Global methylation levels of H3K9 and acetylation levels of H3K56 in IVF and CICT embryos. (**A**) Representative immunofluorescence images of H3K9me2 in embryos at the four-cell and blastocyst stage. Embryos were stained for H3K9me2 (green) and DNA (DAPI, blue). Bar = 100 µm. Quantification of fluorescence intensity at the four-cell and blastocyst stage (*n* = 10 per group). (**B**) Representative immunofluorescence images of H3K56ac in embryos at the four-cell and blastocyst stage. Embryos were stained for H3K56ac (green) and DNA (DAPI, blue). Bar = 100 µm. Quantification of the fluorescence intensity at the four-cell and blastocyst stage (*n* = 10 per group). Labeling intensity was expressed relative to that of the IVF group (set as 100%). The data are from three independent experiments and are means ± SEM (* *p* < 0.05).

The above results indicate that epigenetic markers are differentially expressed in cultured cells. To analyze the effects of these donor cells on cloned embryos, we compared H3K9 methylation and H3K56 acetylation markers among AF-CICT, EC-CICT, and IVF at various developmental stages. Immunofluorescence analysis revealed that the H3K9me2 signal intensity significantly differed between CICT and IVF four-cell embryos (Figure 4A). Similarly, H3K9me2 signal intensity in both EC- and AF-CICT day-8 blastocysts were significantly higher (*p* < 0.05) than that in IVF day-8 blastocysts. Furthermore, a significant difference (*p* < 0.05) was found in the intensities between AF-CICT and EC-CICT blastocysts. H3K56 acetylation signal intensity was also significantly higher in EC-CICT four-cell embryos than in AF-CICT, but not IVF, four-cell embryos (Figure 4B). This shows that EC transfer improves the developmental competence of cloned embryos. Moreover, H3K56 acetylation expression in the EC-CICT day-8 blastocysts were significantly different (*p* < 0.05) from that in the AF-CICT, but not IVF, day-8 blastocysts.

### 2.5. DNA Methylation and Histone Acetylation Levels in IVF and CICT Embryos

**Figure 5:**
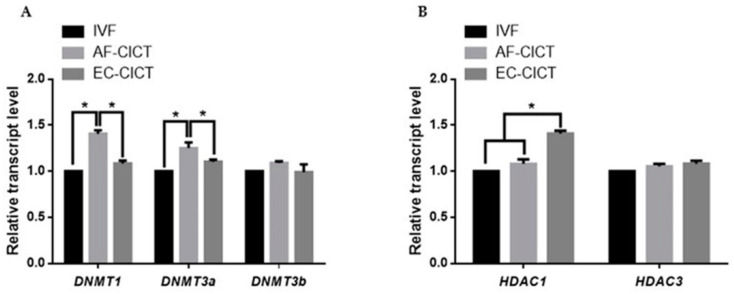
Relative mRNA expression levels of DNA methylation and histone acetylation in blastocysts determined by quantitative reverse transcription PCR. (**A**) Relative mRNA expression levels of *DNMT1, DNMT3a, DNMT3b.* (**B**) Relative mRNA expression of *HDAC1*, and *HDAC3* in blastocysts from the IVF, AF-CICT, and EC-CICT groups. The data are from three independent experiments and are means ± SEM (* *p* < 0.05).

DNA methyltransferases (DNMTs) and histone deacetylases (HDACs) are responsible for epigenetic modifications in bovine IVF and CICT embryos. We analyzed the relative mRNA expression of *DNMT1*, *DNMT3a*, *DNMT3b*, *HDAC1*, and *HDAC3* genes in IVF- and CICT-cloned embryos. As shown in Figure 5A,B, *HDAC1* mRNA abundance was higher in the EC-CICT than in the AF-CICT. However, *DNMT1* and *DNMT3a* expression was lower in the EC-CICT than in the AF-CICT. *DNMT3b* and *HDAC3* expression was not significantly different. These results suggest that aberrant DNA methylation occurs during the early development of bovine CICT embryos.

## 3. Discussion

This study primarily aimed to identify the effects of donor cell types on cloned embryos using CICT. Our results demonstrate the potential for the development of the blastocyst stage of cloned bovine embryos derived from AFs and ECs (Figure S1). We found that EC genomics and epigenetics are more similar and suitable for enucleated oocyte cytoplasm, and additional cytoplasm significantly improved the genomics, epigenetics, and developmental competence of the cloned embryos.

Approximately 23 animal species, including amphibians, fish, insects, and mammals, have been successfully cloned via SCNT. Thus, the SCNT serves as a universal asexual reproductive tool. However, the current state-of-the-art use of SCNT for animal multiplication is questionable. To overcome the drawbacks of SCNT, we used CICT to clone bovine embryos. During enucleation, some of the cytoplasm is lost, the somatic cells are reprogrammed, and embryonic genome activation is delayed. In CICT, the volume of enucleated oocytes is restored by injecting extra cytoplasm from the donor oocyte, thereby improving the efficiency of the cloned embryos [7,8]. However, the shortcomings of SCNT are also barriers to CICT. To address this shortcoming, we attempted to analyze the effects of the donor cell on CICT that were similar to those previously performed for SCNT [13,14,15]. Our results indicate that injecting ECs into enucleated oocytes can stabilize the genetics, epigenetics, and proteomics of developing cloned embryos.

Embryonic genome activation is a basic problem in SCNT-cloned embryos [20]. In this study, we investigated the expression of pluripotent genes, such as *OCT4*, *NANOG*, and *SOX2*, and found that they were significantly and highly expressed in the EC-CICT (Figure 2). The transcription factors *NANOG*, *OCT4*, and *SOX2* are vital regulators of pluripotency and are essential for maintaining the fate and pluripotency of embryonic stem cells. *SOX2* and *OCT4* regulate the pluripotent-specific expression of several genes through a co-operative interaction [21]. Several recent studies have demonstrated that *OCT4* and *SOX2* expression is downregulated in cloned bovine blastocysts compared to their IVF counterparts [22,23,24]. We also found similar results in AF-CICT; however, the expression of these genes was not significant in the IVF and EC-CICT. To examine the quality of bovine blastocysts cloned using CICT, we examined the ICM and the ratio of ICM/TCN in blastocysts, as these two parameters are the criteria for evaluating blastocyst quality [23,25,26]. Embryos containing many cells are likely to implant and develop in the mother uterus [27]. Such findings indicate that the total number of blastomeres was significantly higher in the EC-CICT than in the AF-CICT. The number of apoptotic cells is also a criterion for evaluating blastocyst quality [28]. Embryos with high total cell number and few apoptotic cells are likely to implant and develop [23,29]. The expression of NF-κB, a critical transcriptional regulator of apoptosis, was also assessed [30].

Chromatin modelling plays an important role in embryo genome activation. Therefore, we investigated the expression profiles of chromatin remodeling-related genes. Numerous studies have found that the abnormal modification of histone acetylation and DNA methylation in donor cells may result in failed embryo development using SCNT [31,32,33]. Therefore, we analyzed histone acetylation and DNA methylation levels in cloned bovine embryos derived from different cell types. Our results showed that donor cells markedly affected the epigenetic statuses of cloned embryos. Furthermore, histone acetylation and DNA methylation levels were observed at the blastocyst stage. Global H3K56ac levels were higher in the EC-CICT than in the AF-CICT. Additionally, global H3K9me2 levels were lower in the IVF than in the AF-CICT, while those in the EC-CICT levels were intermediate. These differences are due to the nuclear reprogramming of donor cells, which, in addition to altering DNA methylation patterns, alters histones and other DNA accessory proteins that regulate chromatin [34].

Currently, the accumulated data have revealed that preimplantation-cloned embryos display marked differences in gene expression, affecting the developmental competence of cloned embryos after implantation [35] and potentially inducing high developmental competence in cloned embryos. Therefore, we analyzed histone acetylation and DNA methylation levels in cloned bovine embryos derived from different cell types. The expression of chromatin remodeling proteins (*HDAC1*, *HDAC3*, *DNMT1*, *DNMT3a*, and *DNMT3b*) correlates with the reprogramming efficiency of cloned embryos. Thus, we investigated the effect of cloned embryos on the expression of these chromatin remodeling genes. We found that *DNMT1* and *DNMT3a* expression was significantly lower in the EC-CICT than in the AF-CICT, while *HDAC1* expression was significantly higher in the EC-CICT than in the AF-CICT.

In summary, using ECs as donor cells for nuclear transfer affected apoptosis- and development-related gene expression, could modify global H3K56ac histone acetylation and H3K9me2 DNA methylation, and increased the ICM/TCN ratio. Overall, the ECs can be viable and efficient donor cells for CICT to produce cloned animals and, to a certain extent, improve the development of cloned bovine embryos.

## 4. Materials and Methods

Unless otherwise noted, all chemicals and reagents were obtained from Sigma-Aldrich (St. Louis, MO, USA). All methods and experimental procedures were carried out in strict accordance with the recommendations of the Gyeongsang National University Institute of Animal Care Committee (Approval No. GNU-130902-A0059).

### 4.1. Primary Cell Establishment and Nuclear Donor Cell Preparation

AFs: AFs were prepared as previously described [7]. Briefly, Hanwoo cattle (Korean native cattle) skin tissue was washed thrice with Dulbecco’s phosphate-buffered saline (D-PBS; Gibco, Grand Island, NY, USA), cut into 1 mm^2^ pieces and digested with 0.25% trypsin/EDTA solution (Gibco) at 37 °C for 1 h. The cells were then washed thrice and cultured in Dulbecco’s modified Eagle’s medium (DMEM; Gibco) containing 15% fetal bovine serum (FBS; Gibco), 1% nonessential amino acids, 1% L-glutamine, 100 IU/mL penicillin, and 0.1 mg/mL streptomycin at 37 °C in 5% CO_2_ in air until confluent. AFs at passage 3 were frozen in DMEM supplemented with 10% FBS and 10% dimethyl sulfoxide (DMSO) and stored in liquid nitrogen. The donor cells were thawed and cultured until they became confluent (passages 4–8); thereafter, they were used for cloning.

ECs: ECs were prepared as described previously [36]. AF-CICT derived hatching or hatched blastocysts were cultured on 8-well IbiTreat µ-plates (Ibidi GmbH; Martinsried, Germany) containing 700 µL in vitro culture medium 1 (IVC1) containing Advanced DMEM/F12 (Gibco) supplemented with 20% heat-inactivated FBS (Corning; Corning, NY, USA), 2 mM l-glutamine, 100 IU/mL penicillin and 0.1 mg/mL streptomycin, 1× ITS-X (Invitrogen; Carlsbad, CA, USA), 8 nM ß-oestradiol, 200 ng/mL progesterone, and 25 µM N-acetyl-L-cysteine. After culturing in IVC1 for 2 days, the culture medium was switched from IVC1 to IVC2 (FBS replaced with 30% KnockOut Serum Replacement (Gibco)). After culturing for 10 days, the donor cells for CICT were washed with D-PBS and digested with 0.25% trypsin–EDTA for 3 min. Trypsin activity was blocked using TCM-199 containing 0.3% BSA. Finally, the cell suspensions were allowed to recover at 37 °C for approximately 15 min before nuclear transfer.

### 4.2. Oocyte Collection and In Vitro Maturation (IVM)

IVM was performed as described previously [37]. Hanwoo cattle ovaries were obtained from a local abattoir and transported to the laboratory within 2 h in sterile saline at approximately 35 °C. The ovaries were washed with fresh D-PBS, and the cumulus–oocyte complexes (COCs) were aspirated from follicles (2–8 mm in diameter) using an 18-gauge needle attached to a vacuum pump. The aspirated fluid was expelled into 100 mm Petri dishes containing Tyrode lactate-HEPES (TL-HEPES) medium (114 mM NaCl, 2 mM Na_2_CO_3_, 3.2 mM KCl, 0.34 mM sodium biphosphate, 10 mM sodium lactate, 2 mM CaCl_2_, 0.5 mM MgCl_2_, 1 µL/mL phenol red, 10 mM HEPES, and 100 IU/mL penicillin and 0.1 mg/mL streptomycin) and imaged using a stereomicroscope. COCs with homogeneous cytoplasm and more than three compacted cumulus cell layers were selected and washed in TL-HEPES medium. Thereafter, approximately 50 COCs were sequentially matured in NUNC 4-well plates (Nunc; Roskilde, Denmark) containing 700 µL IVM medium consisting of TCM-199 (Gibco) supplemented with 10% FBS, 1 µg/mL estradiol-17β, 10 µg/mL follicle-stimulating hormone, 10 ng/mL epidermal growth factor, 0.6 mM cysteine, and 0.2 mM Na-pyruvate under 5% CO_2_ at 38.5 °C for 22–24 h.

### 4.3. In Vitro Fertilization (IVF)

Matured COCs were fertilized as described previously [38]. Frozen semen was thawed in a water bath at 37 °C for 1 min, washed, and pelleted in D-PBS by centrifugation at 750× *g* for 5 min at room temperature. The pellet collected from the bottom of the tube was re-suspended in 500 µL 20 µg/mL heparin prepared in IVF medium (Tyrode lactate solution supplemented with 22 µg/mL sodium pyruvate, 6 mg/mL BSA, 100 IU/mL penicillin, and 0.1 mg/mL streptomycin), and incubated at 38.5 °C in a humidified atmosphere containing 5% CO_2_ for 15 min to facilitate capacitation. Subsequently, the spermatozoa were diluted in IVF medium (final density, 1 to 2 × 10^6^ sperm/mL). The matured COCs were transferred to NUNC 4-well dishes containing sperm in 600 µL IVF medium and then incubated in a humidified atmosphere containing 5% CO_2_ at 38.5 °C for 18–20 h.

### 4.4. Nuclear Transfer

After IVM for 22–24 h, the cumulus cells were stripped from COCs by repeated pipetting in 0.1% bovine testicular hyaluronidase prepared in TL-HEPES. Denuded oocytes with first polar bodies were enucleated as previously described [7]. In brief, enucleation was accomplished by aspirating the first polar body and a small amount of the surrounding cytoplasm in TCM-199 microdrops containing 7.5 µg/mL cytochalasin B (CB) and 0.3% BSA. The disaggregated donor somatic cells were immersed in Sendai virus (SV; Cosmo Bio, Tokyo, Japan) solution for 1 min, as indicated previously [39]. In brief, the freeze-dried inactivated SV envelope was combined with 260 µL suspension buffer and diluted to 1:4 with fusion buffer. The donor cells were injected into the perivitelline space of each enucleated oocyte using CICT [7]. The reconstructed embryos were fused using SV-mediated fusion and incubated in SOF+BSA+ITS+EGF medium [27] supplemented with 5 µg/mL CB for 2 h. Successfully reconstructed embryos were activated by incubation in 5 µM ionomycin for 5 min, followed by incubation in 2 mM 6-dimethylaminopurine in a humidified atmosphere containing 5% CO_2_ at 38.5 °C for 4 h.

### 4.5. In Vitro Culture (IVC)

After incubation with sperm for 20 h and/or reconstructed embryo activation, the presumed zygotes/activated embryos were washed extensively and cultured in 600 µL SOF+BSA+ITS+EGF medium [27] in a humidified atmosphere containing 5% CO_2_ at 38.5 °C for 3 days. After checking, 8-cell stage embryos were cultured until day 8 of embryonic development (Day 0 = day of IVF or fusion) in the culture medium. On day 8, the blastocysts were washed thrice in 1 M PBS and stored at 4 °C after fixation in 4% paraformaldehyde until the cells were counted. For gene expression analysis, day 8 blastocysts were transferred to 1.5 mL Eppendorf tubes (five blastocysts per tube), snap-frozen in liquid nitrogen, and stored at −80 °C.

### 4.6. Apoptosis Assays

The apoptotic index of day 8 blastocysts was determined using the TUNEL assay, as previously described [40]. TUNEL assay was performed using an in situ cell death detection kit (Fluorescein; Roche Diagnostics Corp.; Indianapolis, IN, USA) according to the manufacturer’s protocol. Briefly, day 8 blastocysts were washed thrice with 0.3% polyvinylpyrrolidone (PVP) prepared in 1M PBS (PVP–PBS) and fixed in 4% paraformaldehyde overnight at 4 °C. The fixed embryos were permeabilized (0.5% Triton X-100 and 0.1% sodium citrate) at room temperature for 30 min and then washed twice with PVP–PBS. After permeabilization, the embryos were incubated in the dark with fluorescent-conjugated terminal deoxynucleotidyl transferase dUTP at 37 °C for 1 h. Thereafter, the stained embryos were washed in PVP–PBS, incubated with 4,6-diamidino-2-phenylindole (DAPI; 1:100 in D-PBS) for 10 min, washed twice in PVP–PBS to remove the excess DAPI, mounted onto a glass slide, and analyzed to derive their nuclear configuration. The number of cells per blastocyst was determined by counting the DAPI-stained cells under an epifluorescence microscope (Olympus IX71, Olympus, Tokyo, Japan) equipped with a mercury lamp. TUNEL-positive cells appeared bright red, indicating the occurrence of apoptosis.

### 4.7. Immunofluorescence Staining for Epigenetic Markers in Somatic Cells and Embryos

Immunofluorescence staining was performed as described previously [41]. In brief, the donor cells and embryos were fixed in freshly prepared 4% paraformaldehyde solution at room temperature for 30 min, washed thrice in PVP–PBS for 10 min each, and treated with proteinase K (1:1000) for 10 min. For membrane permeabilization, the fixed samples were treated with 0.5% Triton X-100 in PVP–PBS solution for 20 min. Subsequently, they were washed thrice in PVP–PBS and stored in blocking buffer (3% serum albumin + 10% FBS) at room temperature for 1 h. After the samples were incubated with primary antibodies in four-well dishes at 4 °C overnight following washing with PVP–PBS, they were incubated with secondary antibodies (FITC- and TRITC-conjugated, Santa Cruz Biotechnology; Dallas, TX, USA) at room temperature in the dark for 90 min. Finally, the samples were washed and stained with DAPI (1:100 (*v*/*v*) in PBS) for 10 min. Images were viewed under a confocal laser microscope (Olympus, FV1000, Tokyo, Japan). The signal and area were obtained using the ImageJ analysis program to determine the relative integrated density (National Institutes of Health; Bethesda, MD, USA; https://imagej.nih.gov/ij). After normalization, the background intensity was subtracted from each experimental group image. The antibodies used for immunofluorescence analysis are listed in Table S4.

### 4.8. RNA Extraction and Complementary DNA (cDNA) Reverse Transcription

Donor cells and day 8 blastocysts were transferred to a 1.5-mL Eppendorf tube, snap-frozen in liquid nitrogen, and stored at −80 °C. Total RNA was extracted (*n* = 20 per group; four biological replicates with five blastocysts per replicate) using an Arcturus Pico Pure RNA Isolation Kit (Life Technologies, Inc.; Foster City, CA, USA), according to the manufacturer’s guidelines. RNA concentration and purity were determined with a NANO DROP 2000c instrument (Thermo Fisher Scientific; Wilmington, DE, USA). RNA samples were used immediately or stored at −80 °C until further use. The mRNA was reverse-transcribed into first-strand cDNA (Bio-Rad Laboratories Hercules, CA, USA), according to the manufacturer’s instructions. The cDNA was stored at −80 °C until further use.

### 4.9. Quantitative Reverse Transcription PCR (RT-qPCR) Analysis

RT-qPCR was performed in duplicate using a CFX98 real-time system (Bio-Rad Laboratories, Inc.) with a 10 µL reaction volume containing 3 µL diluted cDNA, 0.2 mM of each bovine-specific primer (Table S1), and 1× iQ SYBR Green Super mix (iQ SYBR Green Super mix kit, Bio-Rad Laboratories, Inc.). All cDNA samples were subjected to RT-qPCR using glyceraldehyde-3-phosphate dehydrogenase (GAPDH) primers to detect any variation in internal control gene expression. After confirming the non-significant difference in GAPDH expression among the samples, all transcripts were quantified using independent RT-qPCR. PCR amplification was performed under the following conditions: initial denaturation at 95 °C for 5 min, followed by 44 cycles of 95 °C for 15 s, 57 °C for 20 s, and 72 °C for 30 s, and a final extension at 72 °C for 5 min, and a melting cycle starting from 65 °C to 95 °C with a 0.2 °C/s transition. The 2−ΔΔCt method was used to calculate the target gene expression. For comparison, the average expression levels of each gene from skin-derived cells were set to 1 for donor cells. In contrast, the expression levels from IVF blastocysts were set to 1 for blastocysts.

### 4.10. Statistical Analyses

All data are expressed as the mean ± standard error of the mean (SEM) and were analyzed using Student’s *t*-test or ANOVA. The data were tested for normality and homogeneity of variances using SPSS 18.0 (SPSS Inc.; Chicago, IL, USA) statistical software. The histogram values of fluorescence intensities were measured using ImageJ software (version 1.50, National Institute of Health). Student’s *t*-test was used to analyze the differences between two groups and one-way ANOVA followed by Tukey’s multiple range test, which was used for comparisons among more than two groups. Differences were considered statistically significant at *p* < 0.05.

## Figures and Tables

**Table 1:** Developmental competence and quality of IVF and CICT embryos produced using different types of donor cells.

Groups	No. of Oocytes	No. (%) of Fused Embryos	No. (%) of Cleaved Embryos	No. (%) of Embryos Developed	
				8–16 Cell	Blastocyst
IVF	241	-	205 (85.2 ± 1.5)	186 (77.3 ± 0.9) ^a^	88 (36.6 ± 0.4) ^a^
AF-CICT	275	221 (81.1 ± 1.1)	175 (78.5 ± 2.1)	136 (61.9 ± 3.1) ^b^	65 (29.4 ± 1.2) ^c^
EC-CICT	462	381 (82.2 ± 1.7)	301 (78.8 ± 1.6)	260 (68.6 ± 1.7) ^b^	124 (32.8 ± 0.8) ^b^

^a–c^ Values with different superscripts in the same column are significantly different (*p* < 0.05).

## Data Availability

The data presented in this study are available in article.

## Supplementary Materials

The following are available online at https://www.mdpi.com/article/10.3390/ijms22115841/s1, Figure S1: Derivation and morphology of bovine embryonic cells (ECs), Table S1: Primer sequences for RT-qPCR, Table S2: Characterization of day 8 bovine blastocysts of three groups, Table S3: Apoptotic analysis of day 8 bovine blastocysts of three groups, Table S4: Primary antibodies used for immunofluorescence.

## Abbreviations

CICT	Cytoplasm injection cloning technology
SCNT	Somatic cell nuclear transfer
COCs	Cumulus–oocyte complexes
IVM	In vitro maturation
IVF	In vitro fertilization
IVC	In vitro culture
AFs	Adult fibroblasts
ECs	Embryonic cells
AF-CICT	AF-derived embryos
EC-CICT	EC-derived embryos
ICM	Inner cell mass
TE	Trophectoderm
TCN	Total cell number
cDNA	Complementary DNA
RT-qPCR	Real-time quantitative PCR
