# Supplementary material for: Effects of Donor Cell Types on the Development of Bovine Embryos Using Cytoplasm Injection Cloning Technology
Source: Int J Mol Sci. 2021 May 29;22(11):5841. doi: 10.3390/ijms22115841 (PMC8197982; doi:10.3390/ijms22115841)
Supplement: Supplementary file 1 [file ijms-22-05841-s001.zip › ijms-1183465-supplementary.pdf]

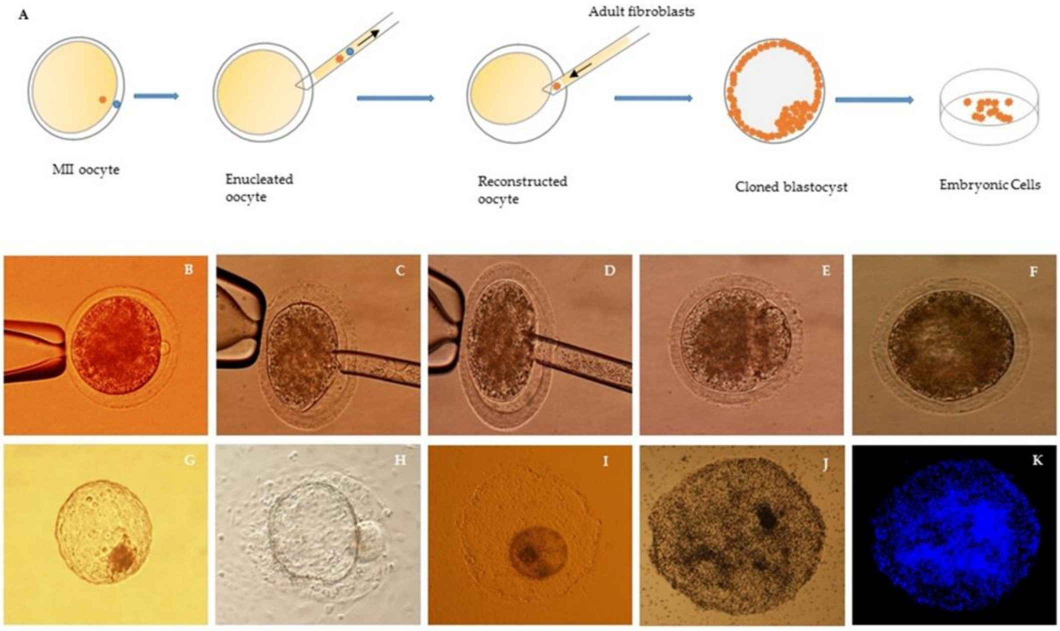

Supplementary Figure 1. Derivation and morphology of bovine embryonic cells (ECs). (A) Strategy for derivation of ECs. By injecting the nuclei of adult fibroblasts (AFs) into enucleated oocytes, reconstructed oocytes were obtained. When the cloned embryos developed to the stage of hatching or hatched blastocysts, the zona pellucida (ZP) was removed and 2-3 blastocysts per culture insert were placed in the 8-well IbiTreat μ-plates. Outgrowths from the attached blastocysts were seen and left to expand until day 5–7. ECs were morphologically large cuboidal cells. (B) Intact oocytes. The MII is pointed by an arrow. (C) Enucleated oocyte. (D-E) The procedure of injecting the nuclei of AFs and cytoplasm into enucleated oocytes. (F) Fused embryos. (G) Hatched embryo. (H-I) Proliferating outgrowths from one hatched blastocyst on the plates. (J) AFs-derived ECs. (K) DAPI staining in the amplified ECs.

Supplementary table 1. Primer sequences for RT-qPCR.

Genes	Primer sequence	Accession no.	Product size (bp)
NANOG	F: CGTGTCCTTGCAAACGTCAT	DQ069776	66
	R: CTGTCTCTCCTCTTCCCTCCTC		
OCT4	F: CAACAACGAGAATCTGCAGG	AY490804.1	153
	R: ATGTGGCTAATTTGCTGCAG		
SOX2	F: GGTTGACATCGTTGGTAATTTATAATAGC	NM_001105463	88
	R: CACAGTAATTTCATGTTGGTTTTTCA		
CDX2	F: GCAAAGGAAAGGAAAATCAACAA	XM_871005	120
	R: GGGCTCTGGGACGCTTCT		
p53	F: CCTCCCAGAAGACCTACCCT	X81704.1	221
	R: CTCCGTCATGTGCTCCAACT		
Bax	F: GGCTGGACATTGGACTTCCTTC	NM_173894.1	112
	R: TGGTCACTGTCTGCCATGTGG		
Caspase 3	F: TACTTTTCCTGGCGAAATGC	NM_001077840	169
	R: TTGCATGAAAAGCAGAATCG		
BCL2	F: TGGATGACCGAGTACCTGAA	NM_001166486.1	120
	R: CAGCCAGGAGAAATCAAACA		
DNMT1	F: AGGGAGACGTGGAGATGCTG	AY244709	194
	R: CATGGAGCGCTTGAAGGAG		
DNMT3a	F: AGACATGTGGGTTGAACCCG	AY271298	188
	R: GGCTCCCACAAGAGATGCAG		
DNMT3b	F: CAGGATGGGAAGGAGTTTGGA	AY244710	151
	R: CACCAAACCACTGGACCCAC		
HDAC1	F: ACCTTTATCCCACAACCCTTCA	NM001037444	201
	R: TCCCTTTTACCCAGTACCCATT		
HDAC3	F: AAGTTTGAGGCTTCTGGTTT	NM0012062431	150
	R: GACTCGGTCAGTGAGGTAGA		
GAPDH	F: CCCAGAATATCATCCCTGCT	NM_001034034	185
	R: CTGCTTCACCACCTTCTTGA		

Supplementary Table 2. Characterization of day 8 bovine blastocysts of three groups.

Groups	No. of blastocysts	No. of total cells	No. of TE cells	No. of ICM cells	ICM:TCN cell ratio
IVF	10	191.7 ± 3.9	144.4 ± 4.4	49.2 ± 1.8a	34.8 ± 1.6a
AF-CICT	10	177.3 ± 0.9	141.2 ± 3.9	30.8 ± 0.9c	21.0 ± 0.7c
EC-CICT	10	187.9 ± 4.9	145.4 ± 5.2	42.5 ± 1.2b	29.7 ± 1.6b

[a-c] Values with different superscripts denotes significantly different within same column ($p < 0.05$). Data are mean ± SEM of at least five independent replicates.

Supplementary Table 3. Apoptotic analysis of day 8 bovine blastocysts of three groups.

Groups	No. of blastocysts	No. of total cells	No. of apoptotic cells	Apoptotic cells rate
IVF	10	194.0 ± 9.9	5.4 ± 0.5[a]	2.9 ± 0.3[a]
AF-CICT	10	172.1 ± 7.5	4.7 ± 0.4[a]	2.8 ± 0.4[a]
EC-CICT	10	190.5 ± 5.2	3.1 ± 0.3[b]	1.6 ± 0.2[b]

[a-c] Values with different superscripts denotes significantly different within same column ($p < 0.05$). Data are mean ± SEM of at least five independent replicates.

Supplementary Table 4. Primary antibodies used for immunofluorescence.

Primary Antibody	REF no.	Company
CDX-2	Am-392	Biogenex
H3K56ac	ab71956	Abcam
H3K9me2	ab1220	Abcam
NF-κB	sc-271908	Santa Cruz Biotechnology
CDK-2	NU-906	Biogenex
CDK-4	PA5-27827	Invitrogen
DAPI	62248	Thermo Fisher scientific
FITC	F-2765	Invitrogen
TRITC	A16071	Invitrogen
